# Topology Control in Spherical 3D Sensor Networks

**DOI:** 10.3390/s26134085

**Published:** 2026-06-27

**Authors:** Nikolaos Zarifis, Dimitrios Katsaros

**Affiliations:** Department of Electrical & Computer Engineering, University of Thessaly, 38334 Volos, Greece; nzarifis@uth.gr

**Keywords:** topology control, clustering, Fibonacci sphere, coverage, connectivity, energy efficiency, 3D wireless sensor networks

## Abstract

The deployment of three-dimensional Wireless Sensor Networks (3D WSNs) in complex environments demands robust topological control to ensure both reliable and fault-tolerant sensing and communication. In order to simultaneously achieve the two objectives over time, an even distribution of the sensors’ energy consumption is essential. Achieving optimal sensor distribution on non-planar surfaces (3D shapes), such as spheres, while maintaining reliable network routes is a significant algorithmic challenge. While many approaches effectively and efficiently addressed the aforementioned goals in 2D environments, and there exists a significant body of work on coverage, connectivity, or energy efficiency in 3D sensor networks, the solutions for either can not straightforwardly be adapted to the 3D case (e.g., some coverage problems are optimally solved for 2D but are still open problems in the 3D case), or the solutions to the individual problems in the 3D case are not integrated gracefully to solve the entire problem. Moreover, these problems have not been address for the realistic spherical 3D case. This paper presents a novel holistic algorithm designed to generate energy-efficient, optimal sensor topologies over spherical 3D sensor networks that guarantee redundant coverage to deal with sensor failures, connectivity with controlled redundancy support for more efficient communication, and the creation of a hierarchy over the flat network to deal with energy issues, at would be appropriate for real-world tasks. The proposed methodology is executed in three primary phases. First, it approaches the geometric part of the problem to determine the optimal placement of sensor nodes on the surface of a sphere, guaranteeing *k*-coverage for the target area. Second, it creates a reliable inner-layer backbone network of sensors that establishes *k*-connectivity ensuring a reliable network for data transmission and distribution of total power in the whole network. Finally, after formulating sensors into clusters, a mathematical formula to change each cluster head is created so that we achieve even distribution of energy consumption across the network. To validate the proposed approach, a 3D WSN software simulator was developed. This tool provides a dynamic visual simulation of the network, enabling the execution, visualization and simulation of the hybrid algorithm and any other 3D WSN.

## 1. Introduction

Wireless Sensor Networks (WSNs) consist of spatially distributed, autonomous devices that cooperatively monitor physical or environmental conditions. One of the most significant problems for their deployment is the topology control, i.e., the calculation of the positions of individual sensors so that they collaboratively are able to effectively monitor the area under consideration and at the same time being able to communicate among them and with one or more external ‘sink(s)’ in order to carry out the assigned task. Such types of problems have been extensively studied in the past for the 2D case, e.g., [1].

While WSN research for optimal solutions in the topology problem has reached its peak in the case of two dimensions, modern critical applications in three dimensions such as underwater acoustic monitoring [2], atmospheric sensing, aerospace exploration, protection of civil infrastructures such as oil refineries, or several military applications [3] present significantly greater challenges by demanding a reliable topology of sensors that guarantees enhanced coverage, enhanced connectivity and energy efficiency.

So, the transition from 2D to 3D WSNs introduces significant geometric, topological, and computational complexities, as spatial volume and non-planar surfaces require more complex deployment strategies to maintain network efficiency.

Due to the complexity and difficulty of this problem, none of the proposed methods so far for the 3D environment developed a holistic solution that can offer: (a) redundant coverage to deal with sensor failures, (b) connectivity with controlled redundancy to support more efficient routing, and (c) hierarchy formation of the sensor network to account for energy efficiency. These three requirements are not independent with each other, but are interweaved in a complex way which requires that the solutions for the individual problems can easily and effectively cooperate. Moreover, spherical coverage due to the nature of wireless transmission is rarely examined in relevant studies and it is also addressed here.

Within this domain, 3D spherical topologies represent a specialized class of networks where sensor nodes are systematically arranged across the curved surface or within the geometric shell of a sphere or hemisphere. Unlike arbitrary 3D volumetric deployments, these topologies must strictly stick to constant curvature and specific spatial constraints. The practical applications of 3D spherical WSNs are vast and critical, including the structural health monitoring of spherical industrial gas and chemical storage tanks, the deployment of defensive radar and sonar dome arrays, the exterior monitoring of deep-sea habitat structures, the establishment of atmospheric or satellite sensor swarms surrounding planetary bodies and more. So, the set of techniques developed within this paper are aiming at exactly addressing all the aforementioned requirements.

### 1.1. Motivation

While much research has been done on flat, 2D WSNs, solving the problem optimally, moving to 3D environments makes it much harder. This difficulty arises because 2D models natively assume flat Euclidean spaces where distance scaling and angular relationships remain uniform. In 3D spherical deployments, the surface’s continuous curvature prevents perfect planar tiling (for instance, standard hexagonal grid structures cannot perfectly enclose a sphere without geometric distortion). Consequently, spatial mapping must take this curvature into consideration, which fundamentally alters the degrees of freedom for signal propagation and relay node placement. For example, foundational studies have extensively explored topology control and optimization, such as the performance analysis for overlay multimedia multicast on r-ary tree and m-d mesh topologies [4], as well as optimizations focusing on the energy-delay trade-off and relay positioning of wireless butterfly networks [5]. While these works provide excellent insights into network efficiency, logical routing, and energy conservation, their underlying topological models are optimized for planar coordinate systems or logically structured grid spaces. If directly applied to a 3D spherical shell, these traditional m-d mesh or tree structures suffer from severe mapping distortions, resulting in irregular inter-node distances, overlapping coverage areas, and heavily skewed energy consumption. The main motivation for this paper is that existing algorithms struggle to do four things at once on complex shapes like spheres: **a.** cover the area, **b.** keep the network connected, **c.** balance battery life and **d.** all these with redundancy for fault tolerance in real applications. Existing solutions generally have three major flaws:

- **Struggling to solve the simple:** Many existing algorithms try to solve the coverage problem optimally in arbitrary shapes and end up being very complex for simpler ones, often producing small error due to greediness or their parameters configuration.
- **The “energy hole” problem:** To send data back to the base, networks rely on inner “backbone” sensors to route the information. However, existing algorithms are not created to simultaneously manage battery life. Even though they generate optimal paths, the inner sensors end up doing all the heavy lifting, so their batteries die much faster than the surface ones. When these core sensors die, the network breaks apart and fails.
- **No complete layered solution:** Right now, algorithms usually only fix one problem at a time. There exist algorithms for one phase of the problem at a time, covering a surface (the geometry problem) or optimally connecting a given topology of sensors or saving energy algorithms but only a few ones with very simple and low complexity algorithms for each phase.

The resulting gap is clear. There is a strong need for an all-in-one approach. Our hybrid algorithm fixes this by splitting the objective into three phases, as mentioned before, and will be analyzed later on. Furthermore, there is a lack of simple to use 3D visualization/simulation tools built specifically to test and visualize any complex 3D WSN as existing visualization libraries of programming languages are not very user friendly to simple users that lack the knowledge of writing complex code. Providing accessible, visual simulation interfaces is increasingly recognized as a crucial factor for enabling autonomous research and learning, paralleling the broader technological push toward automated, user-friendly educational methods [6].

### 1.2. Challenges in Spherical Coverage and Connectivity

The fundamental operational requirements of any 3D WSN are sensing coverage and communication connectivity (for data transmission). In many applications, simple coverage is often insufficient due to the probability of node failure or environmental interference. As a consequence, *k*-coverage is mandated to ensure that every point in the target area (such as the surface of a sphere) is monitored by at least *k* distinct sensors. At the same time, the network must maintain *k*-connectivity within its inner communication backbone. This ensures that the network graph remains connected, providing fault-tolerant, multi-hop routing pathways even if up to k−1 nodes fail. Addressing both these challenges in the same problem requires moving beyond simple geometric or network algorithmic solutions and actually combining them into specialized topological algorithms.

Beyond spatial deployment, one of the most important factors in the operation of 3D WSNs is the finite, sometimes irreplaceable, energy capacity of the sensors (as hardware). In poorly optimized topologies network traffic is distributed unevenly causing routing bottlenecks. Nodes with high centrality value over long periods of time rapidly exhaust their power reserves, leading to network partitioning and reduced overall network lifespan. Therefore, a viable 3D topology control strategy must not only solve the geometric constraints of coverage and connectivity but also guarantee an even distribution of energy consumption across all participating nodes. The algorithm analyzed in this paper is developed for real world applications achieving efficient topologies.

### 1.3. Contributions

This paper contributes by proposing a hybrid algorithm that produces good, for real-world scenarios, topologies for sensors distributes on sphere surfaces. More specifically based on the phase categorization analyzed earlier:

- It suggests the use of Fibonacci spheres for deploying sensing sensors in an outer layer to achieve 3-coverage, and transmission nodes in an inner layer to achieve 3-connectivity. The Fibonacci sphere lattice is highly appropriate for this solution because it provides a deterministic, mathematically uniform, and nearly optimal isotropic distribution of points across a spherical surface, avoiding the polar distortions found in standard grid patterns. This uniformity allows us to calculate the absolute minimum number of sensors required. As a result, we optimize hardware deployment costs and internal energy consumption while maintaining sufficient structural redundancy for real-world fault tolerance.
- It introduces a novel clustering architecture that divides sensors into a dynamically calculated number of clusters for data aggregation, paired with a highly efficient policy for cluster head (CH) rotation. The novelty of this contribution lies in its deviation from traditional probabilistic methods. Instead of relying on random chance to elect CHs, our policy dynamically rotates them based on deterministic hardware parameters (such as the target sphere’s range, and the sensors’ sensing and transmission ranges) combined with the exact topological data acquired during the deployment phase, guaranteeing reliable energy balancing for critical applications.

It also contributes via the development of a simulator for 3D WSNs for developing, visualizing, simulating and testing.

The paper is organized as follows: the related work is presented in Section 2; the mathematical formulation of the problem is given in Section 3, and in Section 4 we provide the details of the proposed Hybrid Topology Generation algorithm. The evaluation of the proposed methods is presented in Section 5, a brief description of our 3D WSN simulator is given in Section 6, and finally Section 7 concludes our work.

## 2. Related Work

### 2.1. Spherical Coverage Techniques

As mentioned, much research has been done on each phase of this problem. Starting from the geometry part we have to answer the questions “How many nodes (sensors) do I have to place? Where (and why)?”. Existing techniques [7,8,9] primarily focus on single-coverage (k=1) while, also, they are focused on optimal placing which is something that leaves gaps in real life applications as external parameters like wind, sea currents, animals and more may interfere with the topology of network. Some efforts focused on *k*-coverage [10], but not on *k*-connectivity.

One of the most optimal methods uses “spherical caps” (curved sensor ranges) to check and find the fewest sensors needed for surface points, but it does not guarantee *k*-coverage [11,12]. Lattice patterns give the best sensor spacing. Triangular grids work best for odd *k* values (like k=3), better over square or hex grids. Even thought they are mainly used on flat areas they can be applied to spheres through density math [13]. Another recent approach uses overlapping spheres to find common covered areas, which could work for surfaces but is built for full 3D space [14]. Some other approaches deal with line-of-sight based coverage models [15] which is not strictly relevant to this paper.

Recent advancements in 3D WSN deployment have also explored AI-driven methods, such as genetic algorithms and particle swarm optimization, to place sensors in full 3D volumetric spaces [7]. While these modern approaches are highly adaptive, they usually focus on free-floating environments (like drones in the air or floating buoys) and struggle to maintain strict, uniform distances when constrained to a hard spherical shell. Unlike these methods, which often suffer from irregular gaps, overlapping densities at the poles, or high computational complexity, the Fibonacci sphere lattice utilized in our approach guarantees a strictly uniform and isotropic distribution of nodes. This geometric property makes it exceptionally appropriate for solving the *k*-coverage problem on spheres with minimal node redundancy.

### 2.2. k-Connectivity on Static 3D WSNs

*k*-connectivity in static 3D Wireless Sensor Networks (WSNs) ensures the network stays linked even after any k-1 nodes fail which is vital for reliable data routing in volumes or surfaces. Most work targets 1-connectivity or planar *k*-connectivity due to search for optimality but, generally, sphere surface specifics are rare.

A study based on statistics and probability for dynamic election of effective routes and connectivity for 3D underwater WSNs balances coverage and connectivity simultaneously [16] but is complicated and has a lot of risk for the sensors as they are placed on the surface of a 3D shape and dynamically switch between sensors for covering and transmitting important data. Another research suggests methods that build *k* separate paths to neighbors for global *k*-connectivity if the full network supports it. EGWO optimizer is used for coverage + connectivity on curves, but not true *k*-connectivity [17].

In the specific context of Underwater Sensor Networks (UASNs), recent topology control studies have introduced depth-based routing and dynamic power adjustment to handle the harsh acoustic environment [18]. These underwater methods focus on fighting severe signal loss and water currents by constantly changing the network’s shape and transmission power. However, constantly adjusting the topology and recalculating routes drains a massive amount of battery. Our approach provides a strong, static baseline topology that avoids the heavy energy cost of constantly moving or recalculating connections.

Generally there do not exist algorithms specifically to handle *k*-connectivity on sphere surfaces after coverage while also maintaining redundancy for real life scenarios. Current 3D work suggest probabilities or fixes and approximations, but they lack efficient static backbone networks for such applications.

### 2.3. Energy Balancing in Static WSNs

Energy efficiency is particularly relevant for underwater sensor networks [19,20], but also in other types of 3D sensor networks. Energy holes occur in static WSNs when some sensors drain their battery faster (e.g., relaying more data), causing early failures and shorter network life. Algorithms aim to spread energy use evenly without moving nodes.

In many static WSNs the backbone network is commonly divided into clusters. Usually a cluster head (CH) is responsible for heavy data aggregation and relaying. This phenomenon is also translated as high energy consumption. There exist many algorithms for CH rotation every certain time spans.

One of the state-of-the-art algorithms for CH rotation uses (usually referring to remaining energy) LEACH algorithm. LEACH picks CHs probabilistically to balance load. Improved versions like ADV-LEACH2 or EEC use static clusters but dynamic CH selection by energy/distance. MST-based methods (Prim’s algorithm) build energy-efficient trees linking nodes shortest paths first, cutting total transmission energy [21].

Furthermore, the HEED (Hybrid Energy-Efficient Distributed) algorithm uses two different rules to choose its cluster heads (CHs). Rather than picking randomly, HEED looks at how much battery life a sensor has left. If multiple sensors have good battery life, it uses a second rule: it checks how easy it is for a sensor to reach its neighbors (like how close they are or how many neighbors it has). HEED does a better job than other methods at spreading out the work and keeping the network alive longer. However, when it first tries to assign the CH roles, it still relies heavily on random chance to make its final choices [22].

Another research suggests hierarchical routing with edge nodes selecting paths balancing energy levels and distance to sinks according to probabilistic rules in order to avoid overuse [23], whereas other methods use network science to perform clustering [24], or genetic algorithms [25,26].

The Intelligent Zebra Optimization Algorithm-based Clustering Protocol (IZOACP) suggests a very simple and efficient CH rotation policy. It integrates the Zebra Optimization Algorithm (ZOA), Gaussian mutation strategy, and opposition-based learning mechanism. These techniques enhance population diversity, prevent premature convergence to local optima, and improve global search capabilities. IZOACP accelerates convergence and optimizes the clustering process, resulting in superior energy consumption control and data transmission performance. Additionally, a dynamic adaptive inter-cluster routing mechanism is designed to balance node [27]. But, once again, the final choice relies in a probabilistic policy. Interesting case studies can also be found in [28].

More recently, researchers have turned to Machine Learning and Deep Reinforcement Learning (DRL) to create highly energy-efficient clustering protocols [29]. These smart algorithms learn the network’s traffic patterns over time to optimize cluster sizes and rotate cluster heads. While these modern AI models achieve excellent energy savings, they require a lot of computing power and training time directly on the sensors, which is often impossible for cheap, low-power hardware in extreme environments.

As seen, most state-of-the-art research relies on probabilistic methods or heavy computational models, which is something that risks the structure of the network, dangerous to use in applications that precision is vital such as military or civil protection ones. This is where our algorithm changes the way of thinking as it generates a policy for CH rotation based on information of the topology of the sensors taken just before, from the previous phase of the algorithm. Our clustering contribution addresses this flaw. By replacing probabilistic CH selection with a purely deterministic rotation policy driven by exact geometric and topological data, our algorithm guarantees reliable energy distribution and predictable fault tolerance, eliminating the critical risks of random network partitioning and heavy computational overload.

## 3. Problem Formulation

Before continuing with the presentation of our algorithm it is very important to formulate the problem. As also mentioned before, we highlight, once again, that we try to solve this topology problem for real-case scenarios. Human defined redundancies are generally accepted. Any of these that are going to happen, will be analyzed on why we do them and why they work.

### 3.1. Network Assumptions and Notation

We consider *N* static sensing sensors deployed on a sphere’s surface of radius *R*. Each sensor *i* has position $p_{i}=\left(x_{i},y_{i},z_{i}\right)$ in spherical coordinates $\left(\theta_{i},\phi_{i},R\right)$ or Cartesian coordinates satisfying $∥p_{i}∥=R$. Sensing radius is $r_{s}$, transmission (communication) radius is $r_{c}$ and this algorithm does not require any assumptions for the relation between sensing and transmission ranges. The backbone network is composed of *M* sensors that only use their data transmission ability and it is divided into *K* clusters. It is important to note that sensors are allowed to be placed on any point of the sphere’s surface, no matter the relation between sensing and sphere’s radius. Table 1 presents the key notation:

#### 3.1.1. The Spherical Geometry Model

The target is the sphere surface $M=\{x\in R^{3}:∥x∥=R\}$. Sensors cover spherical caps, intersections of 3D sensing spheres with the target surface. The surface distance between sensors *i* and *j* uses great-circle distance:

(1) $$d_{surf}\left(i,j\right)=R\cdot \arccos\left(\frac{p_{i}\cdot p_{j}}{R^{2}}\right).$$

#### 3.1.2. Sensing and Communication Model

##### Sensing Range and Coverage

Sensor *i* at $p_{i}$ covers surface points x∈M where Euclidean distance $∥p_{i}-x∥\;\leq \;r_{s}$, forming a spherical cap of angular radius $a=\arcsin(r_{s}/d_{i})$ where $d_{i}=∥p_{i}-o∥$ (O = sphere center). **k-coverage** requires every surface point covered by ≥k sensors:

(2) $$\forall x\in M,\;|\{i:∥p_{i}-x∥\;\leq \;r_{s}\}|\geq k.$$

If the sphere’s radius is extremely greater than a sensors sensing one (R≫r), we may assume that a sensor does not cover a spherical cap piece of a surface rather than simply the area of a circle of radius *r* on top of the sphere’s surface. In many real life scenarios, this assumption may safely be done.

##### Transmission Range and Connectivity

Sensors communicate if $d_{surf}\left(i,j\right)\leq r_{c}$. The *k*-connected backbone graph G(B,E) ensures k-node-disjoint paths between any backbone pair, surviving k−1 failures.

Sensors’ energy consumption mainly follows the formula [30]:

(3) $$E_{i}\left(d\right)=d^{l}+c$$

where $E_{i}\left(d\right)$ is the energy cost of sensor *i* when they want to transmit data in distance *d*. *l* is a constant value expresses how fast the required transmission energy increases as distance grows. When a sensor wants to transmit data in a distance, the density of the material it lies in makes it more difficult to transmit further away. Typically, in the air *l* has value 2 and in water it typically ranges between 2 and 3. *c* is an environmental constant for any other effects the energy cost may get from the environment.

##### Model Realism and Environmental Factors

We must emphasize that in real-world scenarios, actual wireless sensing and transmission signals never form perfect spheres. Environmental factors like weather changes, water currents, physical obstacles, and hardware variations cause signal shapes to become distorted and irregular. In this paper, we use the standard perfect sphere model (the Boolean model) because it provides a reliable, clean mathematical baseline needed to prove our algorithm works theoretically. To bridge the gap to the real world, our algorithm handles these shape distortions by applying a defensive safety margin. Instead of using the maximum possible ranges, the deployment algorithm deliberately shrinks the effective operational ranges (e.g., using 90% of $r_{s}$ and $r_{t}$). This safety scaling forces the sensor nodes to be placed closer together and overlap more tightly, which successfully absorbs real-world signal irregularities and preserves the network’s required coverage and connectivity.

### 3.2. Optimization Objectives and Problem Formulation

The heart of our algorithm consists of the following three phases and we wish to optimize these phase in order to produce clean and accurate results that can serve real life scenarios.

- **Phase 1 (Surface Coverage):** Find positions ${\left\{p_{i}\right\}}_{i=1}^{N}$ minimizing *N* subject to *k*-coverage:
  (4) $$\begin{array}{cc} \; & \min_{\{p_{i}\}\subseteq M}N\\ \; & s.t.\;\forall x\in M,\sum_{i=1}^{N}1(∥p_{i}-x∥\;\leq \;r_{s})\geq k. \end{array}$$
- **Phase 2 (Backbone Construction):** Select the set of *M* dedicated backbone nodes B to maximize the network’s routing *k*-connectivity while minimizing the backbone size M=|B|. Let $\lambda_{k}$ represent the network’s stiffness:
  $$\begin{array}{ccc} \; & \max_{B}\;\lambda_{k}\left(G\left(B\right)\right),\; & \;\;\;\;\;\;s.t.\;G(B)\;\mathrm{is}\;k\text{-}\mathrm{vertex}\text{-}\mathrm{connected} \end{array}$$
  Simultaneously, the physical distance between these transmission nodes must be minimized. This ensures the lowest possible energy consumption for data transmission without unrealistically increasing the required number of backbone sensors *M*. This trade-off is resolved by a mathematical programming model, as shown later.
- **Phase 3 (Energy Balancing):** Design CH rotation policy CH:T→B maximizing network lifetime *T* via uniform depletion:
  (5) $$\begin{array}{cc} \; & \max_{CH(\cdot )}\;T=\min_{i}\frac{E_{i}\left(0\right)}{e_{i}\left(T\right)}\\ \; & s.t.\;\sum_{t=1}^{T}1\left(CH_{t}=i\right)\cdot \mathrm{cost}\left(i\right)\approx \frac{E_{i}\left(0\right)}{\bar{e}} \end{array}$$

In Equation (5), the variable $e_{i}\left(T\right)$ represents the average energy consumption rate of node *i* over the network’s entire lifetime *T*. In physical terms, it measures exactly how fast a specific sensor drains its battery during each operational round. By dividing a sensor’s initial starting energy $E_{i}\left(0\right)$ by its drain rate $e_{i}\left(T\right)$, we calculate the exact time until that specific sensor dies. The main goal of this objective function is to ensure that this battery drain rate is as equal as possible across all relay nodes, which maximizes the overall time *T* before the first failure occurs. Similarly, the variable $\bar{e}$ represents the ideal, perfectly balanced average energy drain rate for the entire network.

This joint problem couples these phases: optimal coverage positions enable efficient backbones, which enable balanced energy policies solved via our hybrid approach.

## 4. Proposed Hybrid Topology Generation Algorithm

### 4.1. Overview of the Hybrid Approach

The hybrid algorithm is divided into three phases. The first phase is the geometric part of the problem: How many sensors will I place and where? The second phase refers to the inner backbone network. Similarly, after placing sensors only for data transmission, who will be connected with who and what clusters will be formed? The third and final phase refers to the creation of a policy that changes the CH of each cluster according to remaining sensor’s energy and distance from cluster’s center. For this algorithm we decided to achieve at least 3-coverage for sensing nodes and at least 3-connectivity for transmission nodes. These numbers apply real-life good redundancy in such networks.

### 4.2. Phase 1: Optimal Surface Coverage

#### 4.2.1. Step 1: Find the Distance in Between the Nodes

This phase of the algorithm begins by first finding the distance the sensing sensors must have in between them. It is going to be distinct into two cases:

- **Case 1** relies on considering the sphere’s surface curvature negligible. In this case ($R\gg R_{s}$), we treat the deployment area as a flat 2D plane and arrange the sensors in a standard triangular lattice. To achieve strict 3-coverage, every physical point must fall inside the sensing circle (radius $R_{s}$) of at least three different sensors. In this flat layout, the hardest points to successfully 3-cover are actually the exact spots where the sensors themselves are placed. If a target stands directly on top of a sensor, it is covered by that sensor. To get two more overlapping coverages, the adjacent sensors must be close enough to reach it. Therefore, the distance *d* between any two adjacent sensors cannot be greater than their physical sensing range. If we set $d\leq R_{s}$, the sensors can “see” each other, and the entire flat grid is perfectly 3-covered. For our evaluation, however, we chose the second case (presented below) to ensure maximum real-world accuracy by fully accounting for the sphere’s curvature.
- **Case 2** is the exact geometric approach taking the sphere’s curvature into consideration for strict 3-coverage. To mathematically guarantee that every single point on the sphere is covered by at least three sensors, we must identify the absolute worst-case boundary points in the lattice. In standard 1-coverage, the hardest point to cover is the centroid of the spherical triangle formed by three adjacent nodes. However, in 3-coverage, the hardest points to cover by three independent sensors are the physical locations of the sensors themselves. If a target is standing exactly at the coordinates of Sensor A, it is covered by Sensor A (distance = 0). To achieve 3-coverage, this target must also fall within the sensing range ($R_{s}$) of at least two adjacent sensors in the lattice. Therefore, the absolute maximum geodesic distance ($d_{arc}$) between adjacent sensors cannot exceed the physical sensing range. If $d_{arc}>R_{s}$, the sensors cannot see each other, and 3-coverage drops to 1-coverage at the vertices. Thus, the exact geometric boundary is elegantly defined as:
  (6) $$d_{arc}=R_{s}$$
  In order to have the minimum number of sensors (optimal hardware cost), we consider $d_{arc}$ as the exact value calculated by Equation (6). However, to leave room for hardware error and geometrical distortions inherent in spherical tiling, we propose taking a conservative effective range of ∼0.9–0.95 of $R_{s}$. In our test cases, 90% of $d_{arc}$ was taken as the effective sensor sensing radius during topological calculation.

**Figure 1:**
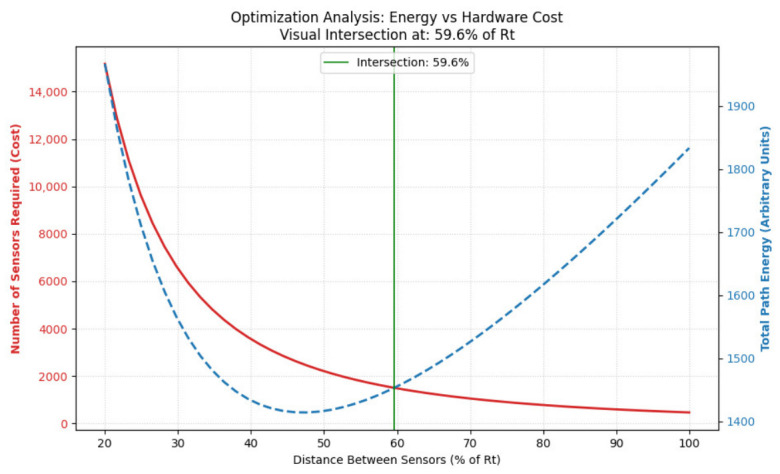
Optimizing sensor distance plot.

**Figure 2:**
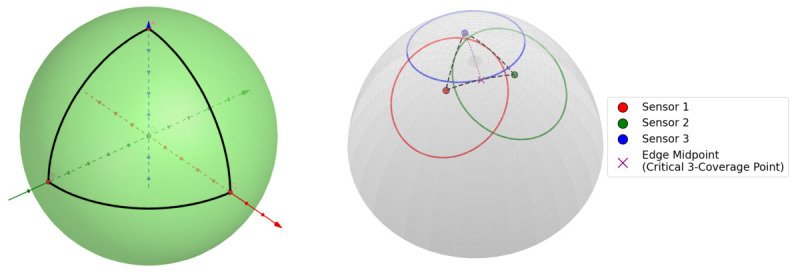
Spherical triangle and coverage target.

Now, we have to find the distance in between the transmission sensors that form the backbone network. As mentioned earlier, this network is a set of nodes used only for data transmission in a small distance in an inner layer of the sphere (not the shell as it was for the sensing sensors but on the shell of a smaller inner sphere with the same center). We want to achieve at least 3-connectivity for redundancy. An optimal solution would be to just place, similarly, the nodes in distance $R_{t}$ in between them but this causes a problem mentioned earlier. The distance is huge so the energy cost rises exponentially. Now, if we place the nodes too close in between them the energy cost to transmit is low but now we have to place a lot of nodes which rises the actual hardware cost. We have to solve the linear programming problem that finds the optimal distance to minimize number of sensors and energy cost. Figure 1 graphically shows the optimal distance to choose for the transmission nodes for a set of realistic values. Similarly this value will be calculated when executing the algorithm. This time, there is no need to test for arc distance on the curvature since *k*-connectivity relies on radio waves, which travel in straight lines through space (Euclidean distance). Therefore, the straight-line distance must simply be less than or equal to $R_{t}$ (the transmission range, not $R_{s}$) to achieve physical connectivity between two relay sensors. Figure 2 [31] shows a 3D implementation of how the sensors form spherical triangles and how we try to achieve 3-coverage on top of target sphere’s surface.

#### 4.2.2. Node Distribution Logic

Now that we calculated the distance sensors must have in outer, sensing shell and inner, transmission layer we may place them onto the surface of target sphere.

Our approach uses the **Fibonacci Sphere** [32] algorithm to evenly distribute the nodes. A Fibonacci sphere takes a number of points as argument and evenly distributes them on the surface of a sphere of a given radius *R*. We calculated the arc distance sensors must have in both layers so, first things first, we will calculate the number of sensors that will actually be needed. Again, here, we have two cases. There are many applications that the sensors’ radius is negligible to the target sphere’s radius and so we may do the assumption that a sensor effectively covers a flat circle’s area. However, because sensor ranges must overlap to avoid blind spots, we cannot simply divide the sphere by the area of a circle. Instead, each node effectively covers a hexagonal tile (comprising two equilateral triangles). In this flat approximation, using the distance *d* calculated in Step 1, the area of this tile is $\frac{\sqrt{3}}{2}d^{2}$. We divide the total surface area ($4\pi R^{2}$) by this tile area to find the required number of nodes. Similarly, we calculate the number of transmission nodes in the inner layer using the total inner surface area divided by the flat tile area created by the optimal transmission distance $d_{trans}$ calculated by the methodology described before and shown in Figure 1.

There are also other applications that cannot assume that the sphere has way larger radius than a sensor’s one and have to take the curvature into consideration. One example can be the optimized deployment of massive satellite-tracking radars around the Earth that have radius’ non-negligible in comparison to Earth’s radius. In this case we have the actual optimized and accurate approach to exact calculation of number of sensors needed. In this case, to calculate the total number of sensor nodes, *N*, required to cover the spherical surface without relying on planar approximations, we must account for the spherical excess inherent in the lattice geometry. To do this, we utilize the exact geodesic distance, $d_{arc}$, that we established in Step 1 as our optimal boundary ($d_{arc}=R_{s\_eff}$). In a regular spherical triangular lattice, the surface area effectively covered by each node corresponds to a Voronoi cell, which is geometrically equivalent to the area of two adjacent equilateral spherical triangles.

First, we define the central angle, θ, formed by the geodesic arc distance, $d_{arc}$, between two adjacent sensors:

(7) $$\theta =\frac{d_{arc}}{R}$$

For an equilateral spherical triangle where all three sides have a geodesic length corresponding to the central angle θ, the internal angle γ is larger than its planar counterpart ($\frac{\pi }{3}$) due to the sphere’s curvature. Using the Spherical Law of Cosines, we calculate γ as:

(8) $$\cos\left(\gamma \right)=\frac{\cos(\theta )}{1+\cos(\theta )}\Rightarrow \gamma =\arccos\left(\frac{\cos(\theta )}{1+\cos(\theta )}\right)$$

According to Girard’s Theorem [33], the area of a spherical triangle is proportional to its spherical excess. If we have a spherical triangle of angles *A*, *B*, *C* on the surface of a sphere of radius *R* then its area is calculated as [33]:

(9) $$T=R^{2}\left(A+B+C-\pi \right)$$

So, the area of a single equilateral spherical triangle is given by $R^{2}\left(3\gamma -\pi \right)$. Since each sensor node in the lattice is effectively responsible for an area equal to two such triangles, the exact effective coverage area per node is:

(10) $$A_{tile}=2R^{2}\left(3\gamma -\pi \right)$$

Finally, to find the minimum number of nodes required to cover the entire sphere, we divide the sphere’s total surface area ($4\pi R^{2}$) by the effective area of a single node tile. The $R^{2}$ terms cancel out, yielding a purely angular, exact formula for *N*:

(11) $$N=\left\lceil \frac{4\pi R^{2}}{2R^{2}\left(3\gamma -\pi \right)}\right\rceil =\left\lceil \frac{2\pi }{3\gamma -\pi }\right\rceil .$$

This formulation is exact for any sphere radius *R* and sensors with sensing range $R_{s}$.

The inner layer has been set to $0.7R_{t}$ inwards the surface of target sphere. Conceptually, this method wraps a continuous spiral from the sphere’s north pole down to its south pole. As the algorithm descends vertically in *N* equal-area steps, it places a sensor node at each step, rotating its horizontal position by the golden angle ($\approx 137.5^{\circ }$). Because the golden ratio is highly irrational, the nodes never perfectly align vertically, spreading them out evenly and closely together. For this deployment, we specifically implement the normalized (or canonical) Fibonacci lattice rather than the classic formula. While the classic approach distributes points efficiently near the equator, it suffers from slight geometric distortion near the poles, causing nodes to either crowd together or leave small coverage gaps. The normalized lattice corrects this by applying a slight mathematical offset to the vertical mapping. This adjustment ensures that the local density of nodes remains constant across the entire surface, guaranteeing a highly accurate and perfectly uniform distribution of sensors even at the extreme spherical caps. More specifically, to generate a spherical lattice, the positions of the points on a spherical lattice, expressed as either longitude + latitude pairs or as 3D coordinates are given as [32]:

(x,y)→(θ,ϕ):(2πx,arccos(1−2y))

and

(θ,ϕ)→(x,y,z):(cosθsinϕ,sinθsinϕ,cosϕ).

**Figure 3:**
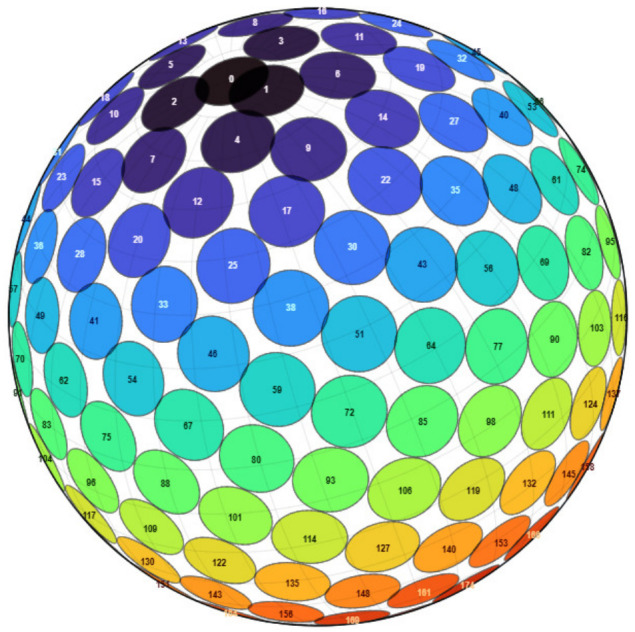
Fibonacci sphere result example.

It is a cylindrical equal-area projection of the square with the final result shown as in Figure 3 [32]. For the purposes of this application which relies on real world problems we convert the coordinates back to Cartesian 3D coordinates (x,y,z) to give the exact positions nodes must be placed according to the target sphere’s center.

This process is done twice for the two layers needed, sensing and transmission.

### 4.3. Phase 2: Inner-Layer Connectivity

#### 4.3.1. Connecting the Inner Spherical Layer

Right now we have placed sensors on two layers of the target sphere but they are not connected, which is the second phase of the algorithm. As mentioned earlier, we want to achieve 3-connectivity for redundancy. A naive approach would be to just connect each sensor to all their possible neighbors. This would achieve 6-connectivity (proved that distances support such a connectivity) but it is not helpful in real applications; as many active links increase energy consumption and, on the actual hardware, if links that cross one another exist, signals tangle in space resulting in false data transmissions, all this without a further benefit. For this reason we suggest a classic, low complexity solution for optimal connectivity, **Spherical Delaunay Triangulation** [34] to **3-connected edge pruning**.

#### 4.3.2. Stage 1: The Spherical Delaunay Baseline

Normally, calculating this mesh is mathematically complex. However, since all our transmission sensors sit exactly on the surface of a sphere, we can use a geometric shortcut: computing the **3D Convex Hull** [35].

The Convex Hull will act like a wrap around the nodes. On a spherical surface, the flat triangular faces created by this wrap mathematically the spherical Delaunay mesh. By using this method, we instantly generate a topology where zero edges cross each other, naturally eliminating spatial interference [35]. It also limits the average number of connections per node to exactly six, providing a clean starting point for the pruning phase which, also, is faster (less complex) than creating a Unit Disc Graph (UDG) model that in a naive approach has a $O(n^{2})$ complexity and using KD-trees for optimization is thrown down to O(nlogn) (same as Convex Hull as we will see further) but becomes very complicated to implement. Furthermore, according to Balinski’s Theorem, the resulting polyhedral graph is guaranteed to be strictly 3-connected, ensuring baseline fault tolerance [36].

#### 4.3.3. Stage 2: Greedy Edge Pruning

While an average degree of 6 creates a strong network, it is highly redundant. Transmitting data to 6 neighbors wastes energy and causes unnecessary collisions. To ensure real-world fault tolerance, our network only strictly requires 3-connectivity.

To safely reduce the node degree from 6 down to the theoretical minimum to achieve 3-connectivity, we apply a greedy edge-pruning algorithm:

1. **Sort by Cost:** Transmission energy grows exponentially with distance. Therefore, we calculate the geometric length of every edge in the Delaunay mesh and sort them from longest (most expensive) to shortest.
2. **Test for Removal:** We select the longest edge and temporarily remove it from the graph.
3. **Connectivity Check:** We run a graph connectivity check to verify if the entire network remains strictly 3-connected. We use the Hopcroft–Tarjan Algorithm which uses a highly optimized Depth-First Search (DFS) to find “articulation points” and “separation pairs” (pairs of nodes that, if removed, break the graph) [37]. This algorithm is the best for ensuring strictly 3-connectivity on a graph.
4. **Decision:** If the network is still 3-connected, the edge is permanently deleted, permanently saving the energy that would have been used to transmit across it. If removing the edge drops the overall connectivity below 3, the edge is deemed a critical bridge and is immediately restored.

By repeating this test for every edge, the algorithm systematically strips away the longest, most energy-draining diagonal links. The final result is a non-intersecting topology that reduces the average node degree to approximately 3.5 to 4.0. This achieves the least possible energy consumption while guaranteeing network survivability.

### 4.4. Phase 3: Establishing Clusters and Paths to CHs

Generally, in WSNs, having every node transmit data is inefficient. Instead, we group the transmission nodes into clusters, where one node acts as the Cluster Head (CH) to aggregate, process and forward data. Since serving as a CH consumes significantly more battery, we implement a dynamic rotation policy to ensure an even energy distribution across the network and maximize total network’s lifetime.

#### 4.4.1. Cluster Formation

To maintain optimal performance regardless of the network’s scale, the algorithm dynamically calculates the number of clusters, setting it to 5% of the total transmission nodes. This specific percentage is widely recognized as an optimal “sweet spot” for energy efficiency, a standard initially established and mathematically proven by foundational protocols such as LEACH [38]. If the percentage is too low, the clusters become physically massive, forcing regular sensors to drain their batteries exponentially faster just to reach a distant cluster head. Conversely, if the percentage is too high, the network creates too many cluster heads, which collectively burn massive amounts of energy forwarding data to the base station. Once the spherical surface is divided into these balanced clusters, the node physically closest to the geometric center of each cluster is designated as the initial CH. This central placement ensures that the initial transmission energy required from the surrounding cluster members is kept to an absolute minimum.

#### 4.4.2. Cluster Head Rotation Policy

Because the CH naturally drains its battery much faster than standard nodes, the algorithm must periodically rotate the CH. To select the most capable node for the next cycle, every node in the cluster is evaluated using a normalized scoring function, *S*. The node achieving the highest score is elected as the new CH:

$$S=\alpha \left(\frac{E_{res}}{E_{initial}}\right)+\beta \left(1-\frac{Dist}{R_{cluster}}\right).$$

This formula balances two critical metrics:

- **Normalized Residual Energy** ($\frac{E_{res}}{E_{initial}}$): Ensures that only nodes with sufficient battery capacity are considered. Normalizing this value (scaling it from 0.0 to 1.0) is vital as it prevents the raw energy values (e.g., Joules) from mathematically overpowering the distance metric.
- **Normalized Proximity** ($1-\frac{Dist}{R_{cluster}}$): Measures how close the candidate is to the cluster’s ideal spatial center, where $R_{cluster}$ is the distance to the furthest node in that specific cluster. A score approaching 1.0 indicates perfect centralization.

#### 4.4.3. Dynamic Weighting

The parameters α and β (where α+β=1) show the inversive relation between energy and location. Rather than using static weights, this formula dynamically adjusts the energy weight α based on the local node density of the cluster:

α=max(0.5,min(0.9,0.4+0.01×nodesPerCluster)).

**Why this approach is best:** In a dense cluster, many nodes will naturally be located near the center. In this case, because distance is less of an important factor, the algorithm heavily favors residual battery life (α approaches 0.9). On the contrary, in a sparse cluster, electing a node as CH that is very far away from the center could force other members to use excessive transmission power to reach it. In such cases, the algorithm shifts the weight back toward spatial position (increasing β) and keeping a perfect balance on the overall network’s lifetime.

### 4.5. Algorithm Complexity Analysis

Now that we have analyzed the algorithm we must discuss its complexity.

We have a centralized algorithm prior to deployment which limits the computational complexity. The process is broken down as following, where *N* represents the total number of transmission nodes:

- **Node Distribution:** Generating the normalized Fibonacci sphere coordinates requires a single iterative pass which means O(N) complexity.
- **Delaunay Triangulation:** Computing the 3D Convex Hull to establish the planar baseline mesh operates in O(NlogN) time (we used the quickhull algorithm).
- **Edge Pruning:** The Delaunay mesh generates approximately E≈3N edges. Sorting these edges by distance takes O(ElogE)=O(NlogN). The algorithm then evaluates each of the O(N) edges for removal, performing an O(N+E)=O(N) graph connectivity check (using the Hopcroft–Tarjan algorithm for triconnectivity) per edge. This gives, in the worst case, a total pruning complexity of $O\left(N\log N\right)+\left(O\left(N\right)\times O\left(N\right)\right)=O\left(N^{2}\right)$.
- **Clustering and Scoring:** Grouping nodes and calculating the localized CH rotation score, *S*, requires O(N) operations.

The overall computational complexity is bounded by the pruning phase at $O(N^{2})$. For a centralized algorithm, execution with quadratic time complexity is highly efficient, allowing the topology to be generated almost instantaneously for large WSNs, as in real applications we realistically refer to a maximum of a few thousand nodes.

## 5. Performance Evaluation and Results

To validate the contributions outlined in our Introduction and address the motivational challenges of 3D spherical WSNs, our evaluation is structured to map our experimental results directly to our proposed solutions. First, to validate our geometric deployment strategy (Contribution 1), we benchmark the required number of sensing nodes against existing algorithms to prove that our Fibonacci lattice approach minimizes hardware cost while maintaining strict 3-coverage. We then reinforce this with a rigorous numerical point-sampling verification and a hardware sensitivity analysis to prove its statistical robustness against real-world parameter variations. Second, to address the “energy hole” problem (Contribution 2), we evaluate the network lifetime and packet delivery metrics to demonstrate how our deterministic cluster head rotation policy outperforms static and direct routing methods. Finally, all simulations and topological visualizations presented in this section were exclusively generated using our custom-developed 3D WSN simulator (Contribution 3), proving its viability as a testing and visualization environment for complex spherical topologies.

To validate the practical efficiency, scalability, and robustness of the proposed hybrid topology algorithm, a series of simulations were conducted. The testing methodology was designed to evaluate the algorithm across different physical scales, verifying both the mathematical models and the computational performance of the network generation.

### 5.1. Simulation Environment and Parameters

Now, it is vital to test the algorithm and produce the numbers expressing its effectiveness. We have already discussed the algorithm’s complexity. It is not necessary to obsess over the exact time taken to compute the topology, because in real-world applications, this calculation happens just once in a lab before placing the physical hardware. Since there is an abundance of time before deployment, taking a few extra seconds (or even minutes) to calculate the perfect topology does not limit the algorithm’s real-world usefulness.

To prove the efficiency of our pre-deployment geometric calculator, we benchmark our required sensor counts against two standard competitor deployment strategies: a deterministic algorithm, polyhedron-based expansion model (Geodesic), and a stochastic algorithm, uniform random deployment (Random). Comparing our Fibonacci approach against these methods provides a clear baseline to see how much physical hardware we can save while still mathematically guaranteeing strict 3-coverage across the entire sphere. To validate this theoretical guarantee, we also deploy a dense 50,000-point virtual sampling mesh to explicitly map coverage holes and calculate exact coverage percentages under simulated hardware degradation.

We will also test the efficiency of the cluster formation and rotation policy in energy distribution. More specifically, we will test the experimental scenarios as in number of data packets delivered, first node death and network partition using different ways of routing as analyzed further later on.

We will test the algorithm across different scales of target spheres and analyze the required number of sensors (both sensing and transmission) based on different hardware capabilities. Therefore, the primary parameters controlling the network generation are the deployment sphere’s radius (*R*), the sensing range of the outer nodes ($r_{s}$), and the transmission range of the inner relay nodes ($r_{t}$). All tests were executed on a standard desktop environment to verify that the $O(N^{2})$ computational complexity remains highly efficient for offline, centralized deployment calculation. It is important to state that the generation of the Fibonacci spheres, the Delaunay triangulation, and the CH rotation policy took a negligible amount of time (≤0.01 s), while almost all processing time was spent on the greedy edge pruning (ranging from milliseconds to minutes depending on the scale of the network).

### 5.2. Experimental Scenarios

The algorithm was evaluated based on three configurations to test its adaptability. The scenarios are built upon the fraction of total target sphere’s range to hardware’s sensing/transmission range (impacts on two different layers). Units (also in simulator) are arbitrary and may be considered as meters/km/miles, etc. whatever user wants, the important is the fraction mentioned above.

- **Scenario A (Micro-Scale):** Represents a dense, localized deployment, such as structural health monitoring on a pressure dome (R=1000, $R_{s}=300$, $R_{t}=400$).
- **Scenario B (Macro-Scale):** Represents a massive planetary or oceanic monitoring network, testing the limits of the algorithm’s scalability (R=1000, $R_{s}=80$, $R_{t}=150$). In both last two scenarios we have fractions $R/R_{s}$, $R/R_{t}>1$.
- **Scenario C (Meso-Scale):** Represents a balanced, standard regional deployment, such as a smart-city or agricultural monitoring dome (R=1500, $R_{s}=150$, $R_{t}=250$). This tests the algorithm’s scaling behavior when the target sphere radius increases independently of the hardware ranges.
- **Scenario D (Transmission-Constrained):** Represents environments with high signal attenuation, such as underwater acoustic networks, where sensing capabilities far exceed transmission ranges (R=1000, $R_{s}=250$, $R_{t}=100$). This forces the algorithm to generate an exceptionally dense inner relay backbone to support a relatively sparse sensing layer.
- **Scenario E (Sensing-Constrained):** Represents environments requiring hyper-granular sensing but with excellent line-of-sight communications (R=2000, $R_{s}=50$, $R_{t}=400$). This pushes the upper boundaries of the algorithm’s node-generation complexity, resulting in a massive sensing layer supported by a highly pruned transmission layer.
- **Scenario F** (Edge Case—Overwhelming Transmission): Represents a scenario where sensors have overwhelmingly large transmission ranges relative to a small deployment sphere (R=500, $R_{s}=100$, $R_{t}=2000$). This verifies the algorithm’s geometric safety constraints. Here we test an extreme fraction $R/R_{t}\approx 0.25$.
- **Scenario G** (Edge Case—Curvature Distortion): Another edge case scenario where the sphere’s surface curvature can affect the achievement of 3-coverage (R=1000, $R_{s}=1350$, $R_{t}=2000$). Here we test an extreme fraction $R/R_{s}\approx 0.75$.

#### 5.2.1. Impact of Sensor Radius on Coverage

The sensing range ($r_{s}$) directly affects the density of the outer shell. The algorithm successfully scales the number of sensing nodes to guarantee complete global coverage without creating massive physical overlaps. As shown in Table 2, our Fibonacci lattice consistently requires a fraction of the sensors needed by random stochastic deployments to achieve 3-coverage, and performs highly competitively against rigid deterministic models. For example, in the micro-scale Scenario A, our algorithm needs only 198 sensors, while the polyhedron-based (Geodesic) model requires 252, and the Random deployment needs around 740 to guarantee the same coverage without blind spots. This gap widens massively in high-density environments. In the sensing-constrained Scenario E, the Random approach requires an impractical 100,000+ nodes because it relies on pure chance, creating heavily overlapping clusters in some areas while leaving random gaps in others. The Geodesic approach performs much better at 26,012 nodes, and our Fibonacci method requires a highly comparable 28,661 nodes. While the Geodesic model holds a slight edge in node count in certain massive-scale scenarios, it is restricted to specific Platonic solid expansions. Our Fibonacci method provides complete continuous scalability for any sphere size while maintaining highly realistic, optimized node counts for real-world applications.

**Figure 4:**
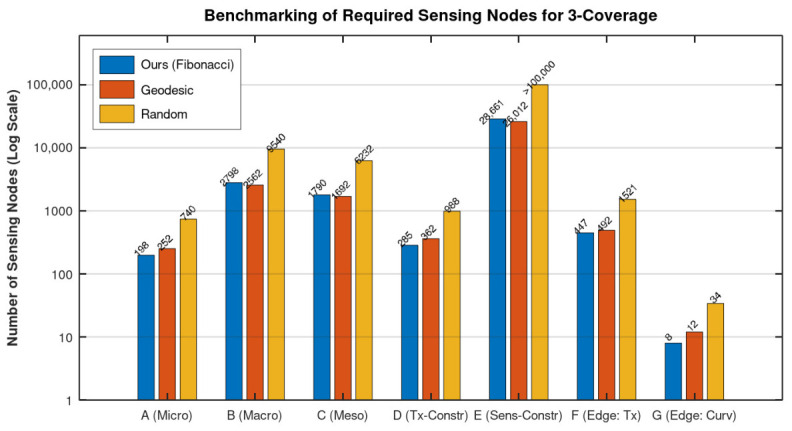
Comparison of number of nodes needed to achieve 3-coverage between algorithms.

What makes these results particularly strong is how the algorithm handles scale. In our standard deployments (Scenarios A and B), the algorithm perfectly maintains the golden 5% rule for cluster heads. For instance, in Scenario B, 209 clusters are formed from 4189 transmission nodes, which is exactly 5%. However, Scenarios F and G act as extreme edge cases. Here, the network is so small that taking 5% of the transmission nodes would result in a fraction of a sensor. Instead of failing or creating an inefficient layout, the algorithm proves its reliability by dynamically defaulting to forming one cluster. This ensures that no matter the scale, from massive macro-networks to tiny micro-deployments, the network architecture remains mathematically stable and physically functional. The plot showing number of nodes used for each of the algorithm is shown in Figure 4.

These results directly align with our first contribution, mathematically validating that the uniform isotropic properties of the Fibonacci sphere allow for highly optimized sensor deployment without sacrificing the strict constraints of 3-coverage and 3-connectivity.

#### 5.2.2. Numerical Coverage Verification and Hardware Sensitivity Analysis

While geometric math is great for theoretical proofs, real-world deployments face unpredictable issues like hardware degradation and signal loss. To prove our network actually works in the real world (and to provide statistical validation), we built a rigorous **Virtual Point-Sampling** tool in our simulator.

We generated a highly dense mesh of 50,000 virtual test points spread evenly across the sphere’s surface. For every single test point, the simulator checked the physical distance to all deployed sensing nodes to verify if it was covered by at least three independent sensors. This allowed us to numerically calculate the exact 3-coverage percentage and easily spot if any blind spots (coverage holes) existed.

To test how tough the network is against hardware failures, we ran a **Hardware Sensitivity Analysis**. We kept the sensors in their exact deployed spots but artificially shrank their physical sensing range ($r_{s}$) from 100% down to 80%. To rigorously prove the algorithm is reliable without running unnecessary, repetitive simulations, we tested this on two specific boundaries: **Scenario A** (our standard, balanced baseline) and **Scenario E** (our most extreme, hyper-dense boundary case). By proving the algorithm survives the absolute toughest conditions (Scenario E), we can confidently say it is robust in all other standard scenarios.

Table 3 shows the results of this test. As expected, under normal conditions (100%), our topology gives a perfect 100% 3-coverage ratio with zero blind spots in both scenarios.

More importantly, the test proves our network is highly resilient to real-world errors. Because we purposely built a 10% safety buffer into our algorithm’s pre-deployment math ($r_{s\_eff}=0.9\times r_{s}$), the physical hardware can fail or lose signal by up to 10% and the network still maintains perfect 3-coverage. It is only when the hardware drops past our built-in safety net (at 88% and below) that blind spots finally start to appear. The fact that this holds true even in the incredibly strict edge-case of Scenario E proves that our algorithm successfully bridges the gap between perfect math and messy real-world deployments.

#### 5.2.3. Operational Boundaries of the Safety Margin

While the static 10% safety buffer ($r_{s\_eff}=0.9\times r_{s}$) mathematically guarantees strict 3-coverage for standard and dense deployments, it is important to define its operational boundaries under extreme spherical curvature.

Through numerical stress-testing, we observed that the heuristic scaling factor begins to experience marginal breakdown in extreme macro-scale scenarios where the sensing range approaches the radius of the deployment sphere ($r_{s}\to R$). For example, in an extreme configuration where R=1000 and $r_{s}=900$, the 0.9 buffer yields a 3-coverage ratio of 99.92%.

This microscopic 0.08% coverage drop occurs due to the inherent packing distortions of the Fibonacci lattice at extremely low node counts (N<30). At this scale, the non-linear spherical excess of the massive triangular tiles outpaces the static linear buffer. Therefore, we define the operational domain of the 0.9 safety margin as highly effective for deployments where the curvature ratio $\frac{R}{R_{s}}\geq 2$. For extreme macro-deployments beyond this ratio, a dynamic or stricter scaling factor (e.g., 0.85) is recommended to absorb the heightened geometric distortion.

#### 5.2.4. Impact of Inner Layer on Network Connectivity

The inner transmission layer ($r_{t}$) affects the multi-hop routing backbone and total clusters number. By applying the second phase of the algorithm, the network actively filters out inefficient radio links. Table 4 shows the efficiency of this pruning process in the inner layer.

The data demonstrates that the algorithm successfully deletes nearly 50% of redundant communication links in normal, large-scale deployments (Scenarios A and B). This is a massive performance optimization. Every active link in a sensor network acts as an open radio channel. By cutting out almost half of these edges, the algorithm drastically reduces radio interference, prevents MAC-layer data collisions, and saves a tremendous amount of battery power that would have otherwise been wasted on redundant listening. We achieve this massive reduction while, also, guaranteeing that alternative routing paths still exist in case a node fails.

Furthermore, scenarios C and D highlight, as before, the algorithm’s way to deal with extreme cases. In these tiny scenarios, the initial network only generated six edges between four transmission nodes. The algorithm correctly did not remove even a single edge as it would break the rules and compromise the network’s layout. Therefore, it stopped the pruning process, resulting in a 0% reduction. This proves that the algorithm does not blindly delete connections just to hit a target metric but balances maximum energy efficiency with guaranteed network survival.

#### 5.2.5. Impact of Cluster Head Rotation on Network Lifetime

Beyond geometric deployment and topological pruning, the survivability of a 3D WSN heavily depends on its dynamic energy management. As established, cluster heads (CHs) bear a disproportionate energy burden due to data aggregation and multi-hop forwarding. To evaluate the effectiveness of our proposed dynamic CH rotation policy (which calculates a normalized score based on residual energy and spatial centrality), we conducted a comparative energy simulation.

The network was evaluated under two configurations: a **Static CH** baseline (where the initially elected CH retains its role until battery depletion causes cluster failure) and our **Proposed Rotation** policy. We define one operational round as the successful delivery of one data packet from every alive sensing node to the sinks. Network longevity is quantified using three critical metrics: First Node Death (FND, indicating the onset of topological degradation), Network Partition (NP, the round where the multi-hop backbone completely fails and sinks become unreachable), and Total Packets Delivered. The same network configuration was used to evaluate each routing method on each scenario.

The results in Table 5 clearly show why having clusters is generally much better than using direct routing. In direct routing, every single sensor acts alone and tries to send its own data across the network to the sink. This creates a massive traffic jam. The nodes located closest to the sink are forced to relay everyone else’s messages, causing their batteries to drain rapidly (the “energy hole” problem). We can easily see this in a realistic, standard network setup like Scenario A ($R=1000,R_{s}=300,R_{t}=400$). Under direct routing, the network delivers only 10,088 packets because the sensors near the sink die very early, leaving the outer sensing nodes disconnected even though they still have plenty of energy. However, when we use clusters, the outer sensing nodes just send their data to a local cluster head (CH), which groups the data together. This allows all the sensing nodes to drain their batteries evenly, quadrupling the total data collected (over 46,000 packets) for the exact same network.

**Figure 5:**
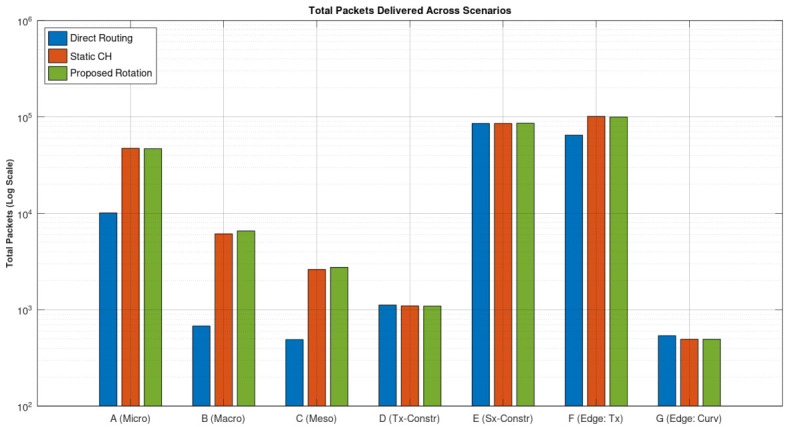
Comparison of efficiency of different routing methods on our topology on total data packets delivered.

However, clustering has a major weakness if it is not managed well. In the **Static CH** approach, one sensor is chosen as the leader and does all the heavy lifting until it dies, which can cause early network failures. Figure 5 and Table 5 prove that our **Proposed Rotation** policy solves this issue by constantly passing the CH role around to the nodes that have the most battery left. By continuously shifting this heavy workload, the proposed method ensures a much more stable network backbone, preventing early total network collapse in critical scaling scenarios. For example, in the macro-scale deployment of Scenario B, the rotation policy more than doubles the time until the network partitions and dies completely (from 17 rounds for static to 39 rounds), while also delivering more data packets. Even in massive sensing deployments like Scenario E, the rotation policy delays the first node death (from two to four rounds) and successfully pushes the total data delivered to over 86,000 packets. While static clustering performs similarly in certain dense edge cases, our dynamic rotation consistently prevents the sudden, early backbone failures seen in standard static deployments.

By outperforming both direct mesh routing (by reducing traffic and preventing sink bottlenecks) and static clustering (by balancing battery use among relay nodes), these metrics directly validate our second contribution. They prove that our deterministic CH rotation policy successfully solves the “energy hole” problem, ensuring a balanced energy depletion that keeps the multi-hop backbone alive longer in large-scale deployments and maximizes the total amount of useful data the network can collect before it ultimately fails.

## 6. Development of the 3D WSN Simulator

Apart from the implementation of the hybrid algorithm, a 3D WSN simulator was also developed. This simulator not only visually displays the outcome from the discussed algorithm but also is used as a general purpose 3D WSN simulator for users to visualize and simulate their own networks and study connectivity, network metrics, etc. The simulator has been implemented as a desktop application for any PC operating system and it is built on top of C++ Qt QML framework.

**Figure 6:**
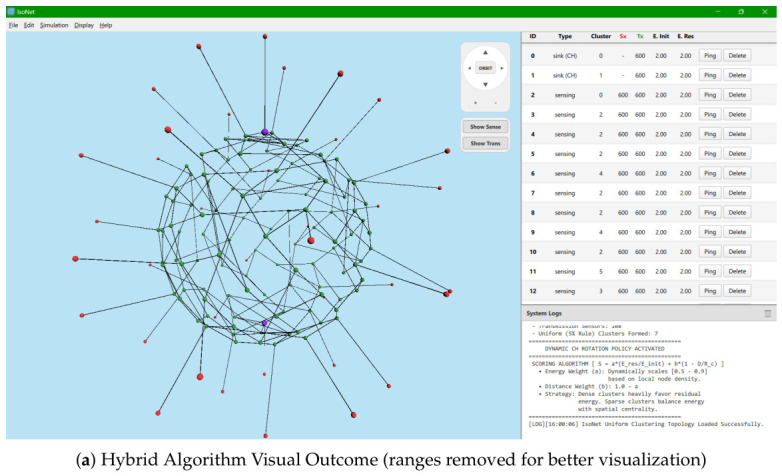

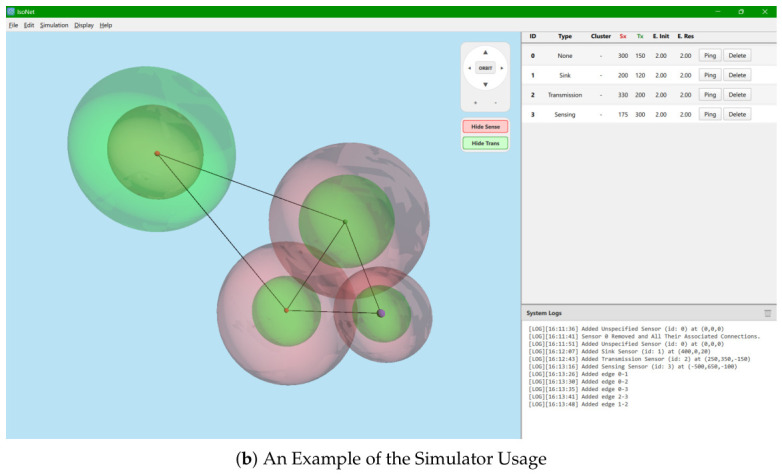
Simulator screenshots.

The simulator, as seen in Figure 6a,b, consists of 3 basic components, on left the 3D view with ability to move, rotate, zoom in/out, display info about sensors etc, on the right top tables with info about sensors, edges, network metrics and the left bottom part which displays logs and information about user’s actions. The simulator software is free and can be found at https://github.com/NikosZ0110/IsoNet/releases/ (accessed at 20 May 2026).

## 7. Conclusions and Future Work

In critical, real-world applications, such as military surveillance, civil protection, underwater base monitoring, or deploying satellite radar networks around a planet and many more, we frequently need to deploy sensors over massive 3D curved surfaces. The core problem this article set out to solve is how to physically place and connect these sensors so that there are absolutely no blind spots, the communication network can survive physical damage or attacks and the batteries drain evenly to maximize the network’s lifespan. Our approach treats the sensor deployment as a deterministic mathematical formula, calculating the exact coordinates and connections before the physical hardware is ever placed.

The deployment of such WSNs in 3D, spherical environments presents significant challenges, especially regarding energy management and radio signal interference. To address these issues, this article introduced an optimized hybrid topology algorithm that solves the entire discussed problem by breaking it up into three main parts.

Through the development and simulation of this hybrid architecture, the following were noticed:

- **Optimal Spatial Distribution of Sensors:** By using normalized Fibonacci spheres, the algorithm guarantees a perfect even distribution of sensors across both layers, solving the k-coverage redundancy requirement.
- **Optimal Sensor Connectivity:** The requirement of 3-connectivity is solved in a simple way and by enforcing it we guarantee real world’s applications redundancy.
- **Balanced Energy Depletion:** The implementation of a dynamic clustering policy (maintaining a 5% cluster head density) proved highly effective. By dynamically adjusting a node’s residual energy weight according to its distance from their cluster center, the algorithm prevents the rapid death of relay nodes, ensuring an even energy drain across the entire network.

While the proposed algorithm is highly efficient, there are a few limitations to the current model:

- **Perfect Spherical Assumption:** The algorithm assumes a perfect spherical surface to deploy nodes. In real-world macro deployments (such as planetary surfaces or terrestrial oceans), arbitrary surfaces like mountains or deep trenches can make direct communication difficult.
- **Homogeneous Range Assumption:** The simulation assumes a stable, uniform transmission range ($r_{t}$) for all nodes. In reality, environmental factors heavily depend on the specific communication medium being used. For terrestrial or aerospace applications using Radio Frequency (RF), factors like weather and electromagnetic interference cause asymmetrical ranges. On the other hand, for underwater acoustic monitoring, electromagnetic waves attenuate too rapidly to be used. In these underwater environments, communication is instead distorted by acoustic absorption losses, multipath effects, and variations in salinity, temperature, and depth (the SSP profile). Other technologies are used by sensors such as, mainly, acoustic waves. This is why a defensive safety margin (using 90% of $r_{t}$) is used to try and solve the problem up to a certain level.

Upon the current implementation, there exist some vital future enhancements that will be done, such as:

- **Different surfaces as topology candidates:** For many on-land applications the wanted shape is a dome or a half sphere. The same algorithm will be implemented for this 3D shape to begin with and later on will be adapted to arbitrary surfaces.

## Figures and Tables

**Table 1:** Key notation.

Symbol	Definition
$S=\{s_{1},\ldots ,s_{N}\}$	Set of *N* sensing nodes
$B=\{b_{1},\ldots ,b_{M}\}$	Set of *M* backbone (transmission) nodes
*k*	Coverage and connectivity redundancy parameter
$\mathrm{CH}=\{ch_{1},\ldots ,ch_{K}\}$	Set of *K* cluster heads (CH⊆B)
$E_{i}\left(t\right)$	Residual energy of node *i* at time *t*
$C_{i}$	Coverage region of sensor *i* on sphere surface

**Table 2:** Benchmarking of required sensing nodes for 3-coverage alongside proposed backbone (based on our algorithm) metrics across expanded scenarios.

	Sensing Nodes for 3-Coverage			Proposed Backbone	
Testing Scenario	Ours (Fibonacci)	Geodesic	Random	Tx Nodes	Clusters
**A (Micro-Scale)**	198	252	∼740	383	19
B (Macro-Scale)	2798	2562	∼9540	4189	209
C (Meso-Scale)	1790	1692	∼6232	3326	109
D (Tx-Constrained)	285	362	∼988	10,051	323
E (Sensing-Constrained)	28,661	26,012	>100,000	2194	73
F (Edge: Overwhelming Tx)	447	492	∼1521	4	1
G (Edge: Curvature)	8	12	∼34	4	1

**Table 3:** Hardware sensitivity analysis and 3-coverage verification (Scenarios A and E).

Actual Hardware Capability	Effective Range (Scen. A/E)	3-Coverage Ratio (Scen. A)	3-Coverage Ratio (Scen. E)	Coverage Holes (Scen. A/E)
100% (Ideal)	300 m/50 m	100%	100%	0/0
95% (Minor Drop)	285 m/47.5 m	99.942%	100%	Detected/0
90% (Safety Buffer Limit)	270 m/45 m	97.938%	98.626%	Detected/Detected
88% (Degradation)	264 m/44 m	94.910%	95.096%	Detected/Detected
85% (Severe Drop)	255 m/42.5 m	87.408%	87.634%	Severe/Severe
80% (Hardware Failure)	240 m/40 m	67.550%	67.148%	Severe/Severe

**Table 4:** Efficiency of the greedy edge-pruning algorithm in reducing transmission layer density.

Scenario	Initial Edges	Removed Edges	Final Edges	% Reduction
Scenario A	1143	485	658	42.43%
Scenario B	12,561	6125	6436	48.76%
Scenario C	9972	4879	5093	48.92%
Scenario D	26,916	11,299	15,617	41.97%
Scenario E	6576	2830	3746	43.03%
Scenario F	6	0	6	0%
Scenario G	6	0	6	0%

**Table 5:** Comparative network lifetime and packet delivery metrics: Direct Routing vs. Static CH vs. Proposed CH Rotation policy.

Testing Scenario	Routing Policy	FND (Rounds)	NP (Rounds)	Total Packets
A (Micro-Scale)	Direct Routing	549	577	10,088
	Static CH	87	554	47,204
	**Proposed Rotation**	**86**	**490**	**46,742**
B (Macro-Scale)	Direct Routing	7	48	677
	Static CH	5	17	6122
	**Proposed Rotation**	**7**	**39**	**6560**
C (Meso-Scale)	Direct Routing	2	37	489
	Static CH	4	12	2615
	**Proposed Rotation**	**4**	**18**	**2750**
D (Tx-Constrained)	Direct Routing	8	9	1118
	Static CH	9	8	1095
	**Proposed Rotation**	**8**	**9**	**1089**
E (Sx-Constrained)	Direct Routing	2	18	85,504
	Static CH	2	17	85,504
	**Proposed Rotation**	**4**	**16**	**86,016**
F (Edge: Over. Tx)	Direct Routing	189	303	64,634
	Static CH	146	303	101,175
	**Proposed Rotation**	**150**	**303**	**99,625**
G (Edge: Curvature)	Direct Routing	51	75	537
	Static CH	38	67	493
	**Proposed Rotation**	**38**	**67**	**493**

**Bold** text indicates our proposed method and the best-achieved performance metrics for each scenario.

## Data Availability

Data available upon request.
